# Point spread function decoupling in computational fluorescence microscopy

**DOI:** 10.1038/s41377-025-02112-5

**Published:** 2026-01-02

**Authors:** Ziwei Wang, Wanyu Gu, Shaolei Xu, Yupei Miao, Zewei Cai, Xiang Peng, Xiaoli Liu, Liwei Liu, Qifeng Yu

**Affiliations:** 1 State Key Laboratory of Radio Frequency Heterogeneous Integration, Shenzhen University, Shenzhen, 518060 Guangdong, China; 2 Shenzhen Key Laboratory of Intelligent Optical Measurement and Detection, College of Physics and Optoelectronic Engineering, Shenzhen University, Shenzhen, 518060 Guangdong, China; 3 College of Aerospace Science and Engineering, National University of Defense Technology, Changsha, 410073 Hunan China

**Keywords:** Biophotonics, Wide-field fluorescence microscopy

## Abstract

Computational fluorescence microscopy constantly breaks through imaging performance through advanced optical modulation technologies; however, conventional theoretical modeling and experimental measurement approaches are challenging to meet the demand for accurate system characterization of diverse modulations. To this end, we propose a point spread function (PSF) decoupling method that is intrinsically compatible with the optimal demodulation in computational microscopic imaging modality. The critical core lies in designing a sample prior-based computational imaging strategy, in which a regular fluorescent sample instead of generally used sub-diffraction limited particles acts as a system modulator to demodulate the system response. PSF consequently can be computationally optimized through the strong support from the modulated sample prior, achieving accurate non-parametric system characterization and thereby avoiding the modeling difficulty and the low signal-to-noise ratio measurement errors of the system specificity. Experimental results across various biological tissues demonstrated and verified that the proposed PSF decoupling method enables excellent volumetric imaging comparable to confocal microscopy and multicolor, large depth-of-field imaging under aperture modulation. It provides a promising mechanism of system characterization and computational demodulation for high-contrast and high-resolution imaging of cellular and subcellular biological structures and life activities.

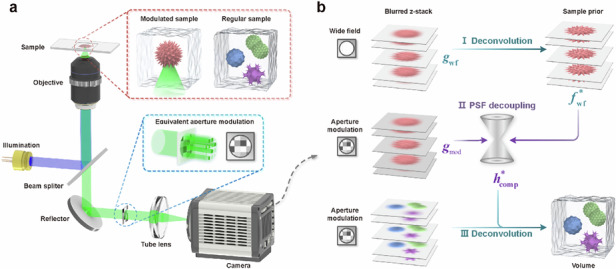

## Abstract (web summary)

Sample priors enable point spread function decoupling in computational fluorescence microscopy, improving system characterization and imaging quality.

## Introduction

Computational fluorescence microscopy (CFM) deeply integrates the molecular specificity of fluorescent proteins with optical modulation and computational demodulation, enabling advanced microscopic observation that plays a significant role in life sciences, medical diagnostics, and materials research^1–3^. Representative technologies, including super-resolution fluorescence imaging such as structured illumination microscopy^4–7^ and single-molecule localization microscopy^8–11^, multi-modality fluorescence imaging that combines quantitative phase imaging^12,13^ and optical diffraction tomography^14,15^, and fluorescence volumetric imaging of computational optical sectioning microscopy^16,17^ and light field microscopy^18–25^, constantly break through the performance constraints of imaging resolution, speed, field of view, and depth.

Accurate system characterization is crucial to improve the observation resolution and fidelity for biological tissues and cells during optimal demodulation in CFM. For instance, for blind deconvolution (BD)^26–28^, point spread function (PSF) prior deconvolution^29–33^, and recently emerging deep learning deconvolution^34–36^ in computational optical sectioning microscopy, accurate PSF can significantly enhance the image contrast and resolution. There are two main categories of PSF obtainment: theoretical calculation and experimental measurement. Theoretical PSF (tPSF) is calculated through imaging modeling informed by known parameters involving numerical aperture (NA), medium refractive index, and excitation/emission wavelength^37–39^. Generally, an actual microscopic system does not entirely conform to the defined theoretical assumptions, especially for computational microscopy through active optical modulation such as light field illumination^19,40^ and aperture modulation^21,22,25^, making it difficult to solve an analytical PSF that matches the system specificity accurately. Alternatively, measured PSF (mPSF) generally uses sub-diffraction limited particles for imaging to obtain the empirical system response^41–43^. However, the detection signal of such microsize particles decays rapidly with the increasing imaging depth, leading to low signal-to-noise ratio (SNR) and measurement deviation. Moreover, the particle model has difficulty providing feedback on the sample specificity related to actual biological sample structure and medium disturbance effect.

In this work, we propose a sample prior-based PSF decoupling method suitable for optical modulation and optimized demodulation in CFM modality. Instead of theoretical modeling and direct measurement, we design a computational imaging strategy that first uses a regular fluorescent sample as an optical modulator to pre-modulate the system response of CFM. The wide-field deconvolution result of the modulated sample is then used as a sample prior to computationally decouple the system PSF from the modulated response signal. This computational PSF (cPSF) has three advantages: (1) compared to BD, which alternatively optimizes PSF and volume data, the strong support from the wide-field sample prior allows for accurate PSF decoupling from the pre-modulated signal; (2) PSF decoupling adapts to diversified optical modulation inherent to CFM and accurately characterizes the non-parametric system specificity, avoiding the modeling difficulties and errors of the microscopic system; (3) it breaks through the low SNR limit when using sub-diffraction limited particles and carries the sample-specific information under the interaction with the sample modulator, enabling high-fidelity object structure restoration. We experimentally demonstrated the comparison results of CFM under aperture modulation, where cPSF could provide high-contrast, high-resolution imaging of subcellular structures across various biological tissues. In high-NA and multicolor experiments, cPSF-based deconvolution also exhibited superior volumetric and large depth-of-field (DOF) imaging with annular apertures. Therefore, the proposed PSF decoupling method facilitates CFM for outstanding observation of cellular and subcellular morphological change and life activity.

## Results

### PSF decoupling in CFM

**Fig. 1:**
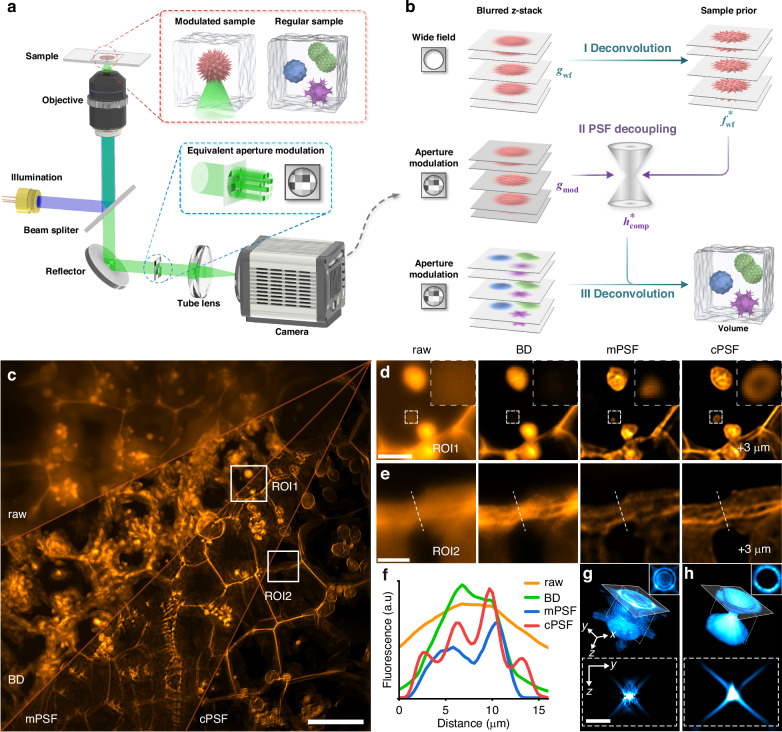
PSF decoupling and deconvolution. **a** Schematic diagram of CFM, the system response can be altered through the aperture modulation of the equivalent pupil to optimize imaging performance; **b** principle and workflow of PSF decoupling, by using a fluorescent sample as a modulator and taking its wide-field deconvolution result as a sample prior, the system PSF can be computationally optimized from the detection signal and finally applied to the deconvolution of any observed fluorescent samples; **c** MIP of *z*-stack of a potato tuber sample; **d,** **e** local enlarged views of *x*-*y* slices at different ROIs, comparing the raw image and deconvolution results using BD, mPSF, and cPSF, respectively; **f** cross-sectional intensity distribution curves related to regions marked by white dashed lines in (**e**); 3D profiles along with central *y*-*z* and marginal *x*-*y* slices of (**g**) mPSF and **h** cPSF. Scale bars, 100 μm (**c**), 20 μm (**d**), 15 μm (**e,** **g,** **h**)

The imaging process of fluorescent samples under excitation illumination can be modeled as: $g=h\otimes f$, where $\otimes$ denotes the convolution operation, $h$ is the system PSF, $f$ is the original object information, and $g$ is the degraded image signal after passing through the imaging system. Deconvolution techniques can be used to restore information from blurred images. For wide-field microscopy, the classical microscopic imaging theory can provide a reliable PSF prior, thereby achieving a sound deblurring performance (Supplementary S1). In comparison, for computational microscopy with active optical modulation, such as the aperture modulation of equivalent pupil demonstrated in Fig. 1a, it is generally difficult to perform accurate theoretical modeling for the modulated system response. In this situation, sub-diffraction limited particles are commonly used as ideal point sources to experimentally detect the system response, i.e., ${h}_{{\rm{meas}}}={g}_{{\rm{sub}}}$, but the low SNR issue of the detection signal would consequently be transferred to the observed PSF.

We use a regular fluorescent sample instead of sub-diffraction limited particles as an optical modulator to pre-modulate the imaging system. At this point, the detection signal is essentially the interaction result of the imaging system with the modulated sample and thus simultaneously contains information on the system PSF and sample modulator. Recovering the system response from the modulated signal becomes feasible, provided a reliable prior of the sample modulator being prepared in advance. To this end, we design a computational imaging strategy for PSF decoupling, as illustrated in Fig. 1b (refer also to the workflow in Supplementary S2). Specifically, the modulated sample (Fig. S1d) is first imaged and deconvolved in wide-field microscopy:

1 $${f}_{\mathrm{wf}}^{\ast }\leftarrow {f}_{k+1}=\left\{\left[\frac{{g}_{\mathrm{wf}}}{{h}_{\mathrm{theo}}\otimes {f}_{k}}\right]\otimes {\rm{B}}({h}_{\mathrm{theo}})\right\}{f}_{k}$$

where ${h}_{{\rm{theo}}}$ is the wide-field tPSF, ${g}_{{\rm{wf}}}$ is the wide-field image, ${f}_{k}$ is the sample estimation at *k*-th iteration, and B(∙) represents the backpropagation. The object information of the deblurred *z*-stack ${f}_{{\rm{wf}}}^{\ast }$ can provide the computational sample prior to PSF decoupling. Subsequently, the modulated sample is re-imaged in computational microscopy, and finally, the system PSF ${h}_{{\rm{comp}}}^{\ast }$ can be computationally optimized from the modulated signal ${g}_{{\rm{mod}}}$:

2 $${h}_{\mathrm{comp}}^{\ast }\leftarrow {h}_{k+1}=\left\{\left[\frac{{g}_{\mathrm{mod}}}{{h}_{k}\otimes {f}_{\mathrm{wf}}^{\ast }}\right]\otimes {\rm{B}}({f}_{\mathrm{wf}}^{\ast })\right\}{h}_{k}$$

where *h*_*k*_ is the PSF estimation at *k*-th iteration.

The high-SNR detection signal, inspired by the modulated sample, provides a computational sample prior, whose strong support guarantees the PSF decoupling accuracy. In contrast to CFM, wide-field microscopy is more easily modeled, with both traditional fluorescent microspheres and PSF models effectively characterizing the system, enabling high-quality sample priors that support subsequent PSF decoupling. During PSF pre-modulation and decoupling, there is no need for parametric characterization of the imaging system, consequently avoiding the modeling error and the adverse effect on subsequent deconvolution. PSF decoupling implicitly contains the modulation characteristic of computational microscopy or the possible aberration and disturbance caused by optical path mismatch in conventional microscopy. cPSF simultaneously provides a flexible non-parametric characterization for the system specificity and carries the biological structure and medium information of the sample modulator, ultimately facilitating the high-fidelity restoration of object information.

Figure 1c–h shows the experimental results using a 20×/0.75 objective lens under annular aperture modulation (Method). The three-dimensional (3D) profile and two-dimensional slices of mPSF obtained using 500nm-size fluorescent microspheres are shown in Figs. 1g and S2a, respectively. It can be seen that mPSF exhibits a low-SNR state, and substantial scattered signals emerge around the main structure. The central *y*-*z* slice reveals a rapid attenuation in mPSF energy distribution with the increasing imaging depth. Despite employing a high-sensitivity sensor with a long exposure time to improve the image SNR, photon signal transmission of sub-diffraction limited particles still diminishes quickly as imaging depth increases, leading to the limitation of effective depth range and PSF accuracy (Fig. S3a–S3c). In contrast, using a modulated sample has high adaptability and flexibility for high-SNR signal detection and accurate PSF decoupling (Figs. 1h and S4b). The cPSF distribution demonstrates an extended depth range and a well-defined profile with a ring-like shape in lateral dimension and a hollow cone-like shape in 3D space, which, in theory, is consistent with the PSF of an annular aperture (Fig. S5b).

Figure 1c–e shows the maximum intensity projection (MIP) of the *z*-stack and local enlarged views of *x*-*y* slices at different regions of interest (ROI) of a potato tuber sample with a thickness of 20 µm, respectively, where the raw image and the deconvolution results using BD, mPSF, and cPSF are compared. Due to the lack of prior constraints on PSF, BD struggles to coordinate PSF estimation and signal reconstruction, resulting in poor adaptability and performance for CFM. In comparison, the cPSF-based deconvolution benefits from accurate PSF decoupling and has the best contrast and resolution. For example, it can clearly resolve the starch granule on the tuber surface (secondary local enlarged view in Fig. 1d) and the multilayer cellular structure of the phloem (Fig. 1e). Figure 1f plots the cross-sectional intensity distribution curves related to the regions marked by the white dashed lines in Fig. 1e. It demonstrates that cPSF enables to distinguish four-layer structure, but mPSF confuses adjacent layers and only resolves two.

### Optical modulation in CFM

**Fig. 2:**
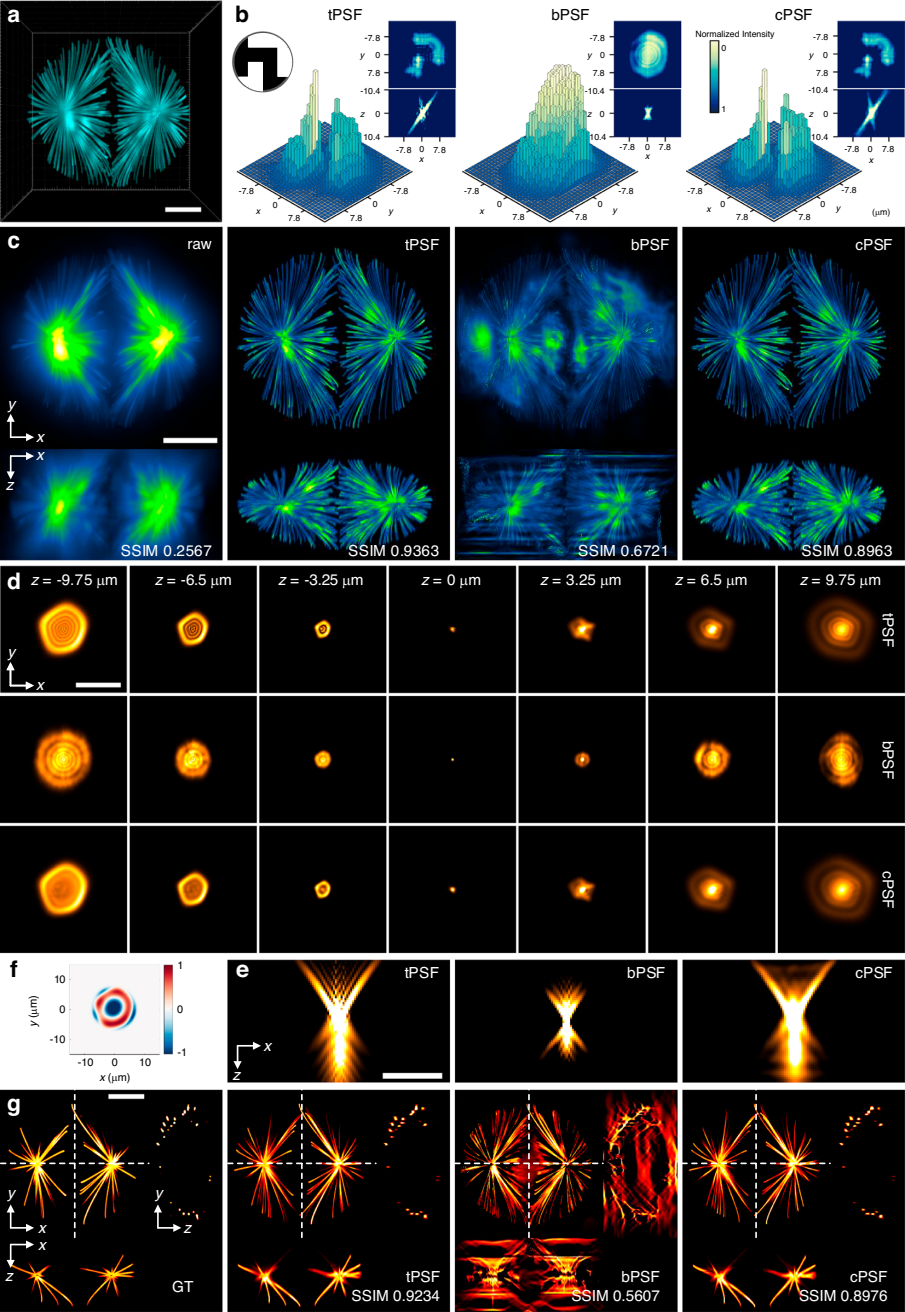
Optical modulation and computational demodulation in CFM. **a** Simulated sample model from DeconvolutionLab2^31^; **b** marginal *x*-*y* slices and their 3D intensity distributions and central *x*-*z* slices of tPSF (as reference), bPSF, and cPSF, and **c** MIPs of blurred *z*-stack and deconvolution results under random amplitude modulation; **d** representative *x*-*y* slices at different axial positions; **e** central *x*-*z* slices of tPSF, bPSF, and cPSF; **f** the corresponding aberration distribution, and **g** central slices of original sample and deconvolution results in the presence of optical aberrations induced by phase modulation. Scale bars, 50 μm (**a**), 20 μm (**c,** **g**), 15 μm (**d**), 10 μm (**f**)

We simulated the imaging process with an open-source sample model^31^ as ground truth, as shown in Fig. 2a, to investigate the influence of optical modulation and aberration on PSF decoupling and deconvolution in CFM. For the convenience of comparison and analysis, the aperture region of the effective pupil was divided into a 4×4 grid and amplitude modulation was applied by randomly gating each subregion, as shown in the upper-left inset in Fig. 2b. Here, tPSF of computational microscopy served as a reference, as shown in Fig. 2b. It exhibits a nontraditional system response that the PSF distribution is obviously asymmetrical, shown by the marginal *x*-*y* slice and its 3D intensity distribution. In addition, the energy distribution of the central *x*-*z* slice has an apparent directional inclination. Blind PSF (bPSF) and cPSF were obtained through BD and PSF decoupling, respectively. In this case, the solution of BD is highly ill-posed and prone to local optimum, resulting in bPSF deviating considerably from the ground truth. In contrast, cPSF is quite consistent with the reference. Figure 2c compares the raw image and the deconvolution results using tPSF, BD, and cPSF, with the structural similarity index measure (SSIM) related to the ground truth of 0.2567, 0.9363, 0.6721, and 0.8963, respectively. It can be seen that BD brings serious artifacts, although deblurring is completed to a certain extent, while cPSF-based deconvolution obtains the closest result as tPSF.

The possible optical path mismatch in microscopy, such as the wear of objective lens and the misalignment among various optical components, and the scattering disturbance from the observed samples may lead to imaging aberration and thus the deviation of the system response from the theoretical model (Fig. S3f). We introduced phase modulation into a wide-field microscope to implement the aberration simulation (Fig. 2f). Figure 2d, e shows representative *x*-*y* slices at different axial positions and central *x*-*z* slices of tPSF (as reference), bPSF, and cPSF, respectively. The presence of optical aberration obviously disrupts both axial and lateral PSF symmetry, and the contours of *x*-*y* slices are no longer regular circles. The proposed method successfully reveals these aberration-induced deviations by accurately computing the distorted PSF. As shown by the comparison of the deconvolution results using tPSF, bPSF, and cPSF with the ground truth, optical aberration may introduce severe artifacts in BD, while the system and sample specificities of PSF decoupling enable cPSF to mitigate the potential adverse effects, achieving high-fidelity volumetric imaging (Figs. 2g, S5d, and S5g).

### Performance comparison of CFM

**Fig. 3:**
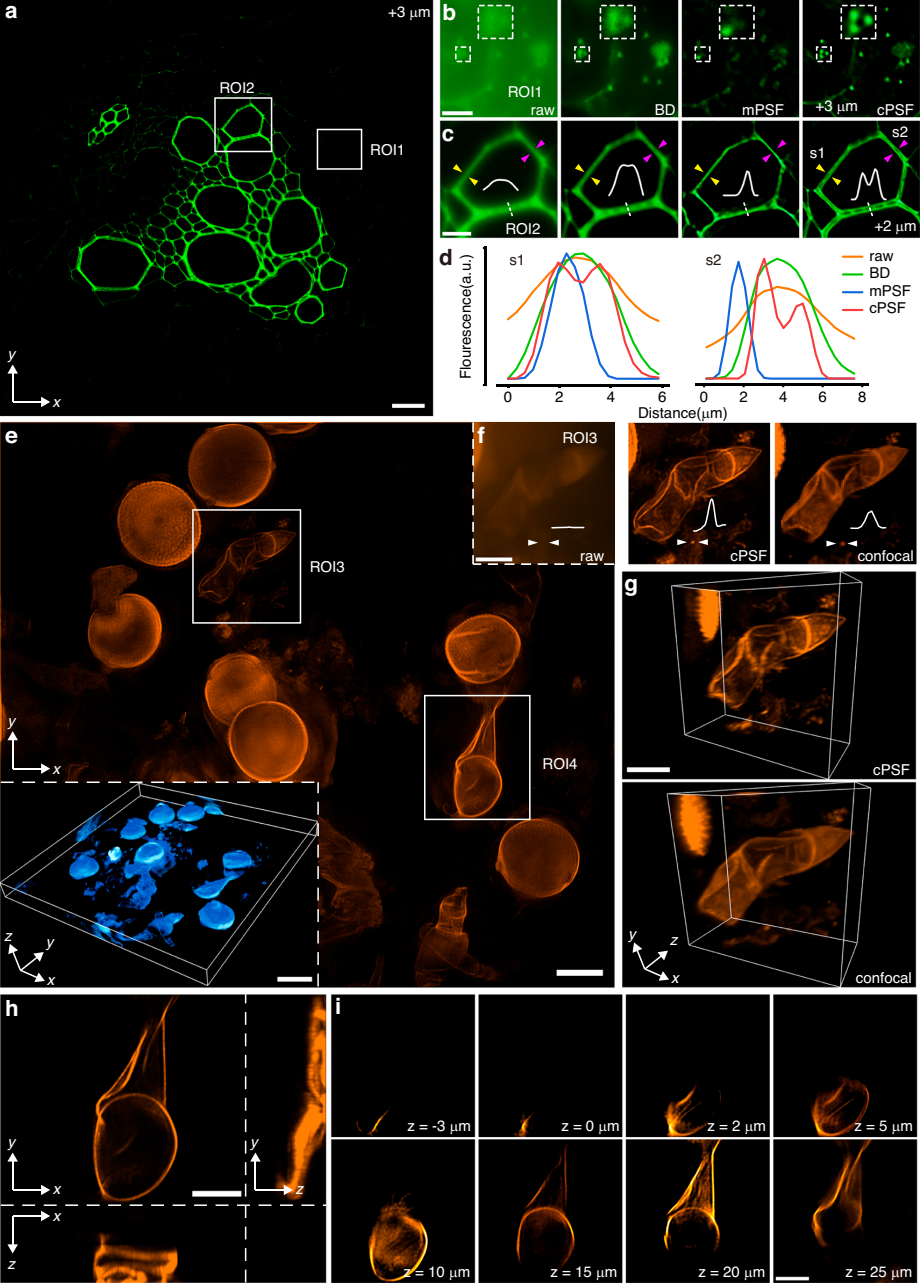
Performance comparison in CFM. **a** *x*-*y* slice of the cPSF-based deconvolution result of a sunflower stem sample; **b,** **c** local enlarged views of *x*-*y* slice at different ROIs, comparing the raw image and deconvolution results using BD, mPSF, and cPSF; **d** cross-sectional intensity distribution curves related to regions marked by yellow and magenta arrows in (**a**); **e** MIP and 3D view of the cPSF-based deconvolution result of a zucchini pollen sample; **f,** **g** local enlarged views of MIP and the corresponding 3D views for comparison with confocal microscopy, respectively; **h,** **i** 3D MIP and depth-resolved *x*-*y* slices of an individual pollen grain, respectively. Scale bars, 50 μm (**a,** **e**), 20 μm (**b,** **c**), 30 μm (**f**–**i**), 100 μm (3D view in **e**)

We further evaluated the performance of CFM using the same microscope setup as that in Fig. 1c. A sunflower stem sample with a thickness of 9 µm was imaged, of which the vascular bundle is presented by the *x*-*y* slice of the cPSF-based deconvolution result in Fig. 3a. Figure 3b, c shows local enlarged views of the *x*-*y* slice at different ROIs to compare the raw image and the deconvolution results using BD, mPSF, and cPSF, respectively. Tiny crystalline particles, of which the fluorescence signal is relatively weak and generally submerged in the bright background of the xylem, are distributed along the medullary rays around the vascular bundle. Compared with BD and mPSF, cPSF-based deconvolution can restore these particle signals located in dark regions in high contrast. The secondary local enlarged views in Fig. 3b illustrate the noticeable differences: submersion (raw image), confusion (BD), one particle (mPSF), and all three particles (cPSF). Additionally, the protoxylem has a fine hollow vessel structure and exhibits high contrast because of fluorescence sensitivity. Even then, the deconvolution results using BD and mPSF can only yield confusion and single-layer structure, respectively (Fig. 3c). In contrast, cPSF-based deconvolution can high-resolution distinguish the double-layer vessel structure, as evidenced by the cross-sectional profiles related to the regions marked by the white dashed lines and the intensity distribution curves (Fig. 3d) related to the regions marked by the yellow and pink arrows in Fig. 3c.

For thick samples, such as pollen, since wide-field illumination will uniformly excite all fluorophores throughout the entire volume, the accompanying out-of-focus fluorescence signal may cause a noticeable background blur, making it difficult to resolve the desired sample details. Figure 3e shows the experimental result of a zucchini pollen sample with a thickness of 50 µm, demonstrating the excellent deblurring performance of the cPSF-based deconvolution through MIP and 3D view. With accurate PSF decoupling, the proposed method achieves optical sectioning performance comparable to laser scanning confocal microscopy (Method) to observe the fine sample structure clearly. For instance, it allowed the recovery of starch granules that had been completely submerged in the background signal of the raw image, as shown by local enlarged views (white arrows) and 3D views in Fig. 3f, g, respectively. This sample contains various morphological states of pollens during their germination process. Figure 3h, i shows the 3D MIP and depth-resolved *x*-*y* slices of a representative pollen grain, respectively, whose target structural information has all been faithfully restored to the intrinsic layers. Additional performance comparison using a mouse brain section sample (Supplementary S7) further validates the robustness and applicability of our proposed method.

### High-NA CFM

**Fig. 4:**
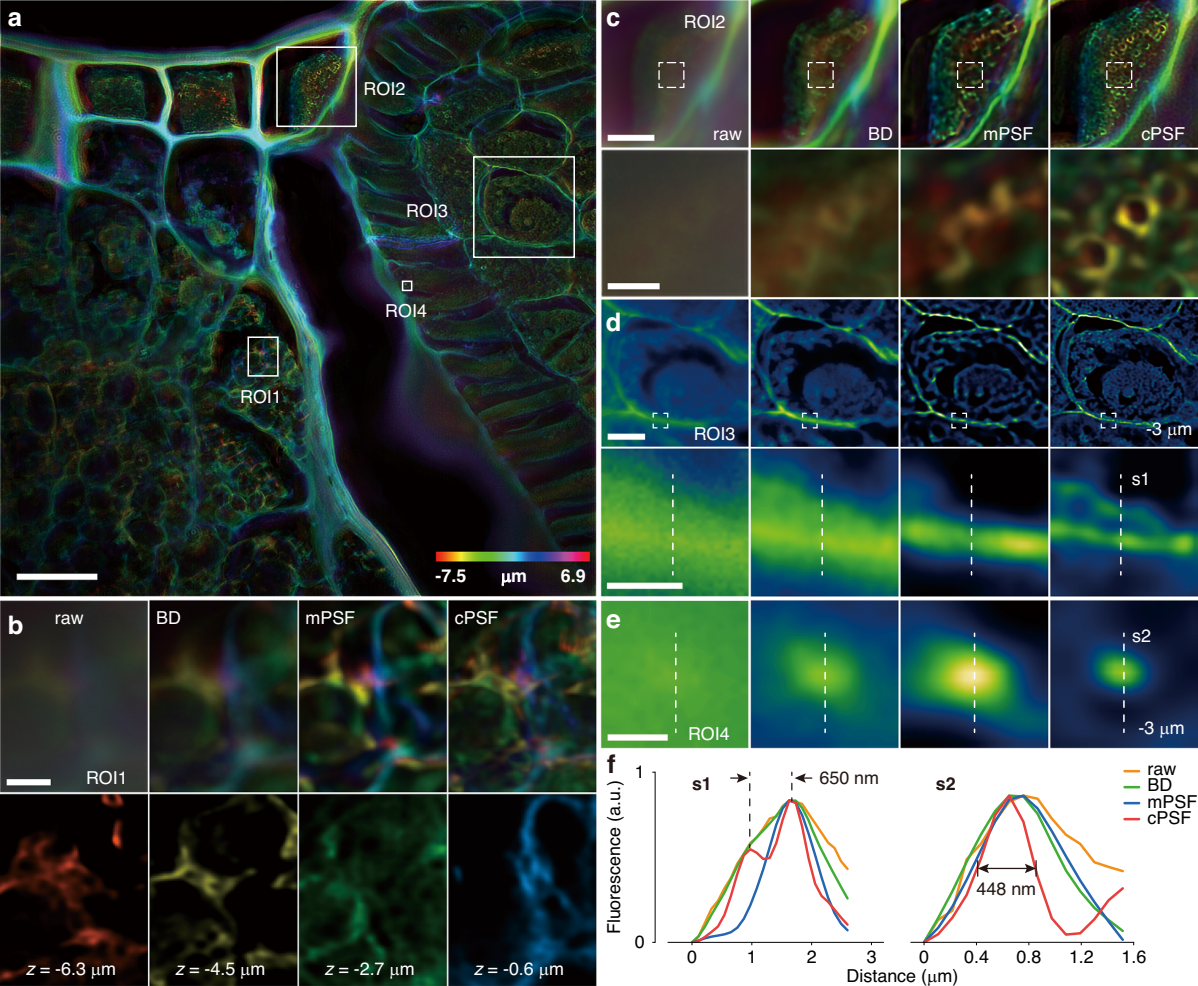
High-NA CFM results. **a** Pseudo-color depth map of the cPSF-based deconvolution result of a wheat seed sample; **b** local enlarged views of the depth map along with *x*-*y* slices of the cPSF-based deconvolution result at different axial positions; **c** primary and secondary local enlarged views of the depth map; **d,** **e** primary and secondary local enlarged views of *x*-*y* slices at different ROIs, respectively; **f** cross-sectional intensity distribution curves related to regions marked by white dashed lines in (**d**) and (**e**), comparing the raw image and deconvolution results using BD, mPSF, and cPSF. Scale bars, 30 μm (**a**), 3 μm (**b**), 10 μm (first rows in **c, d**), 2 μm (second rows in **c,** **d**), 1 μm (**e**)

We employed a 60×/1.2 water-immersion objective lens and the annular aperture modulation with different parameters to further observe finer sample structures (Method). Figure 4a presents a pseudo-color depth map of the cPSF-based deconvolution result of a wheat seed sample with a thickness of 14.4 µm. The endosperm in wheat seeds consists primarily of starch granules, proteins, and a small amount of fat, in which the object layers have relatively obvious changes and complex structures, as shown by local enlarged views of the depth map and *x*-*y* slices of the cPSF-based deconvolution result at different axial positions in Fig. 4b. Using cPSF can eliminate fluorescence signal interference among object layers to the greatest extent and effectively restore the details of the multilayer structures. Both BD and mPSF-based deconvolution fail to separate the aliasing signals from different object layers. In addition, the application of cPSF allows for clear restoration of ring-like structures in the wheat seed sample, as shown by the primary and secondary local enlarged views of the depth map in Fig. 4c. Furthermore, the primary and secondary local enlarged views of *x*-*y* slices in Fig. 4d demonstrate the sharply defined topographic structure of plumule and a 650 nm forked gap (Fig. 4f) related to the regions marked by the white dashed lines from cPSF-based deconvolution. However, the fine plumule structure cannot be resolved or is lost when using BD and mPSF. The proposed method can also resolve the starch granule with full width at half maximum of 448 nm from the raw fluorescence signal that is completely blurred, whereas the relevant results with BD and mPSF are 767 nm and 725 nm, respectively. This distinction is clearly demonstrated by the local enlarged views of *x*-*y* slices and the intensity distribution curves related to the regions marked by the white dashed lines in Fig. 4e, f, respectively.

### Multicolor and large-depth-of-field CFM

**Fig. 5:**
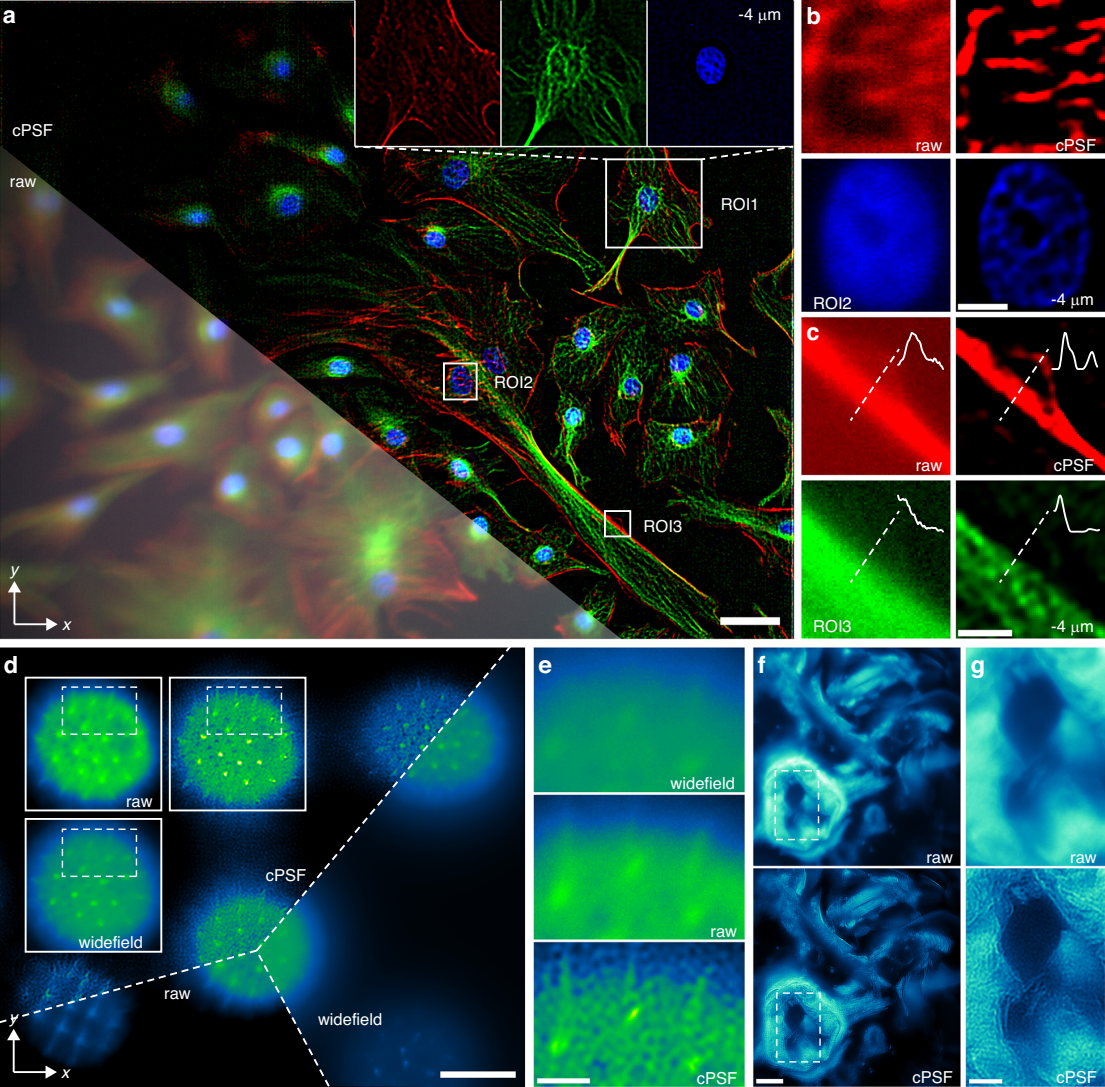
Multicolor and large-depth-of-field CFM results. **a** *x*-*y* slice along with local enlarged views of the cPSF-based deconvolution result of a bovine pulmonary artery endothelial cell sample; **b,** **c** local enlarged views at different ROIs, showing multichannel fluorescence signals of actin (red), microtubule (green), and nucleus (blue); **d** focal plane images and cPSF-based deconvolution result of a pumpkin pollen grain sample under wide-field microscope and CFM; **e** local enlarged views related to regions marked by white dashed wireframes in (**d**); **f** focal plane images and cPSF-based deconvolution result of a crab larva sample under annular aperture modulation; **g** local enlarged views related to regions marked by white dashed wireframes in (**f**). Scale bars, 50 μm (**a,** **d,** **f**), 8 μm (**b,** **c**), 10 μm (**e**), 20 μm (**g**)

Multicolor fluorescence microscopy under annular aperture modulation was performed using excitation illumination with different wavelengths. PSF decoupling across fluorescence channels first was analyzed, showing that a single-channel cPSF could be applied for CFM across broadband spectrum in the experiment (Supplementary S8). Figure 5a shows the *x*-*y* slice of a bovine pulmonary artery endothelial cell sample with a thickness of 20 µm, where the local enlarged views demonstrate that the cPSF-based deconvolution can effectively recover the actin (red), microtubule (green), and nucleus (blue) of the cell. Figure 5b provides detailed enlargements of actin and nucleus in different color channels. The nucleus is the core of cellular life activity, while the actin is wrapped around the microtubule to constitute the entire cytoskeleton, as shown in Fig. 5c. Additionally, the proposed method is also capable of resolving microfibrillar scaffold structure and hemicellulose of a wheat leaf sample (Supplementary S9). Consequently, accurate PSF decoupling combined with deconvolution enables CFM to discriminate and recognize various cell shapes and structures, facilitating the observation of cellular and subcellular morphological change and life activity of biological samples.

The DOF of fluorescence microscopy is generally minimal; for example, the theoretical DOF of the used wide-field microscopy is approximately 2 µm. The DOF extension is crucial for deeper and clearer microscopic observation and can be achieved through annular apertures^25,44,45^. In this experiment, we used CFM under annular aperture modulation to image a pumpkin pollen grain sample. The pollen grain is spherical with a diameter of approximately 100 µm and a surface covered with prominent spikes. Figure 5d compares the focal plane images obtained by wide-field microscopy and CFM, showing that the latter contains more object information spanning an extended DOF. Then, simple image deconvolution using cPSF was performed to suppress the blurring effect to a certain extent, as shown by the local enlarged views in Fig. 5e. Figure 5f, g shows the focal plane blurred image and local enlarged view for a crab larva sample with a thickness of 40 µm, respectively, which are compared with the cPSF-based deconvolution result. These examples verify that the proposed PSF decoupling method can also be applicable to optical modulation-based DOF extension in CFM.

## Discussion

CFM combines specific system modulation with corresponding computational demodulation to gain the desired imaging functions and performance metrics. This modality, which broadens the degree of freedom of manipulation and application space of fluorescence microscopic imaging, has attracted growing attention and study. Accurate characterization of the modulation system is a necessary and critical task to guarantee performance optimization for CFM. However, with the diversification of modulation parameters and the complexity of modulation means, it becomes increasingly challenging to characterize the system through conventional methods such as theoretical modeling and experimental measurement. For example, unpredictable optical path mismatch in an imaging system can be regarded as some randomly occurring system modulations, in which case the system specificity cannot be accurately described. In this work, we propose an inspiring perspective on computational system characterization, revealing that computational imaging strategies can enable conventional fluorescent samples to modulate first and then demodulate the system response, inherently encompassing the system specificity of CFM and the sample specificity of the sample modulator.

Using a regular sample modulator for PSF decoupling overcomes the issues of low SNR and application limitation using sub-diffraction limited particles, expanding the selection range and diversity of sample references for system characterization. Traditional methods, such as the use of fluorescent microspheres or PSF models in wide-field microscopy, are well-suited for reliable fitting and provide high-quality sample priors, ensuring accurate PSF decoupling. Currently, we selected those modulated samples with high-contrast fluorescence features, which were imaged within the field of view to avoid boundary artifacts and reduce the computational burden and error in the PSF decoupling process. A modified sample prior is also necessary as supporting conditions to participate in the optimization computation of the system PSF. In fact, the optimization property of PSF decoupling can reduce the dependence on the sample prior to a certain extent. The influence of sample specificity on PSF decoupling and deconvolution has been discussed (Supplementary S10). It reveals that system specificity dominates the core PSF structure, while sample specificity contributes to fine adjustment for performance optimization.

Although the CFM used for experimental demonstration is diffraction-limited, the proposed framework of PSF decoupling provides a general strategy of accurate system characterization for diversified imaging modalities. In future research, we will design more advanced computational imaging strategies to relax the dependency conditions on modulated sample features and priors, enabling more flexible adaptation to modulated microscopic imaging systems and biological imaging environments, such as super-resolution and dynamic live-cell imaging. Ultimately, it provides a promising mechanism and method of system characterization and demodulation for multi-dimensional manipulation and high-performance breakthroughs in CFM.

## Materials and methods

### System setup

CFM system was constructed with a wide-field fluorescence microscope (IX81, Olympus) equipped with interchangeable objective lenses (UPLSAPO 20×/0.75, 40×/0.9, and 60×/1.2, Olympus). A 4 f relay system (LA1417, f = 150 mm, Thorlabs) was integrated at the imaging port of the microscope, where a custom-fabricated annular mask was placed at the Fourier plane (i.e., the conjugate pupil plane) to perform aperture modulation. The obstruction ratios, defined as the ratio of the inner to the outer diameter of the annular aperture, were set to 0.8 (Fig. 1 and Fig. 3), 0.5 (Fig. 4), and 0.65 (Fig. 5), respectively. An sCMOS camera (C11440-42U40, 6.5 μm × 6.5 μm, 2048 × 2048 pixels, HAMAMATSU) was used for image acquisition. A laser scanning confocal microscope (TCS-SP8, Leica) with an APO 20×/0.7 objective lens was used for experimental comparison. Fluorescence excitation/emission wavelengths were configured as 360 nm/460 nm, 490 nm/530 nm, and 550 nm/620 nm, respectively. All data computation and processing were conducted on a computer workstation equipped with a 72-core Intel Xeon Gold 5220 CPU and an NVIDIA TITAN RTX GPU with 4608 CUDA cores.

### Sample preparation

The biological samples used in experiments included potato tubers, sunflower stems, and wheat seeds (Vic Science & Education) stained with tetramethylrhodamine (TRITC), zucchini pollen grains (SAGA), pumpkin pollen grains (DAKE Education), and crab larvae labeled with DAPI, FITC, and TRITC for multicolor fluorescence imaging.

Bovine pulmonary artery endothelial cells (F14781, Thermo Fisher) were subjected to multiplex fluorescent labeling: F-actin was stained with Texas Red-X phalloidin, microtubule was labeled using a mouse monoclonal anti-bovine α-tubulin antibody (236-10501) in combination with a BODIPY FL-conjugated goat anti-mouse IgG secondary antibody, and nucleus was counterstained with DAPI.

Fluorescent microspheres (T14792, Thermo Fisher) with a particle diameter of 500 nm were employed, incorporating four types of fluorescent dyes with emission peaks at 365/430 nm (blue), 505/515 nm (green), and 660/680 nm (red).

Simulation experiments were performed by setting a 3D voxel grid of 256 × 256 × 128, with a spatial sampling interval of 325 nm, ensuring compliance with the Nyquist sampling criterion. NA and emission wavelength were set to 0.75 and 570 nm, respectively. A publicly available dataset from DeconvolutionLab2^31^ was employed as the test sample. A synthetic sample composed of spherical and rod-like structures was used for PSF decoupling.

### Algorithm setup

Images were presented using image processing and visualization softwares, ImageJ and Imaris. PSF was extracted from an image stack of fluorescent microspheres using the microscopic image processing software, Huygens. In the overall workflow (Supplementary S2), the steps involving deconvolution are modular and interchangeable. The 3D reconstruction software AutoQuant was used due to its strong semi-blind recovery performance (Step 1). Subsequently, the open-source algorithm DeconWolf^33^ was employed in the deconvolution procedure due to its support for custom PSF input and consistently high-quality output (Step 3). Any trusted deconvolution tool and algorithm can substitute for the corresponding module. For instance, alternatives such as the open-source DeconvolutionLab framework^31^ have been tested and are fully compatible with our method.

## Supplementary information

Supplementary

Supplementary Video 1: The scale in supplementary Video 1 is 100 μm.

Supplementary Viedo 2: The scale in supplementary Video 2 is 10μm.

Supplementary Video 3: The scale in supplementary Video 3 is 100 μm.

Supplementary Video 4: The scale in supplementary Video 4 is 10 μm.

## Data Availability

The data and code can be made available upon request.
