# Supplementary material for: Point spread function decoupling in computational fluorescence microscopy
Source: Light Sci Appl. 2026 Jan 2;15:26. doi: 10.1038/s41377-025-02112-5 (PMC12757601; doi:10.1038/s41377-025-02112-5)
Supplement: Supplementary file 1 — Supplementary [file 41377_2025_2112_MOESM1_ESM.pdf]

Supplementary Information for

Point spread function decoupling in computational fluorescence microscopy

Ziwei Wang[1,2], Wanyu Gu[1,2], Shaolei Xu[1,2], Yupei Miao[1,2], Zewei Cai[1,2,*], Xiang Peng[1,2], Xiaoli Liu[1,2,*], Liwei Liu[1,2], and Qifeng Yu[1,2,3]

[1]State Key Laboratory of Radio Frequency Heterogeneous Integration, Shenzhen University, Shenzhen 518060, Guangdong, China

[2]Shenzhen Key Laboratory of Intelligent Optical Measurement and Detection, College of Physics and Optoelectronic Engineering, Shenzhen University, Shenzhen 518060, Guangdong, China

[3]College of Aerospace Science and Engineering, National University of Defense Technology, Changsha, 410073, Hunan, China

* zeweilinc@foxmail.com

* lxl@szu.edu.cn

S1 Fluorescence microscopic imaging and deconvolution

In fluorescence microscopy, the detected image signal $g(x,y,z)$ can be modeled as the convolution of the sample $f(x,y,z)$ with the system PSF $h(x,y,z)$, such that

$$g(x,y,z)=h(x,y,z)\otimes f(x,y,z) \quad \text{(S1)}$$

Ideally, PSF at the focal plane is denoted as $h(x,y,0)=\delta(x,y)$. Accordingly, the detection signal at a specific depth $z=z_1$ can be expressed as:

$$\begin{aligned} g_{z_1}(x,y) &= h(x,y,0)\underset{x,y}{\otimes} f(x,y,z_1)+\sum_{z\neq z_1} h(x,y,z_1-z)\underset{x,y}{\otimes} f(x,y,z) \\ &= f(x,y,z_1)+\sum_{z\neq z_1} h(x,y,z_1-z)\underset{x,y}{\otimes} f(x,y,z) \end{aligned} \quad \text{(S2)}$$

The acquired image thus simultaneously contains in-focus object information (first term) and out-of-focus contributions affected by the system response diffusion (second term), leading to image blurring.

Considering the noise in the actual detection signal, a Poisson imaging model can be established:

$$g(x,y,z)=\mathcal{P}\left[h(x,y,z)\otimes f(x,y,z)+b(x,y,z)\right] \quad \text{(S3)}$$

where $b(x,y,z)$ denotes the background signal and $\mathcal{P}(\cdot)$ denotes the Poisson process. Therefore, due to the diffusion effect of system response and the influence of detection noise, the actual detection signal is a degraded image produced by the imaging system.

The object information can be recovered from the detection signal through deconvolution operation. This process represents an ill-posed inverse problem that typically requires the system PSF as prior knowledge for optimization solution. Gibson and Lanni established a classical PSF diffraction model[1]:

$$h(x,y,z)=\left|\int_0^1 J_0\left(\frac{kN_a\rho\sqrt{x^2+y^2}}{M}\right)\exp\left[ik\phi(z,\psi)\right]\rho\mathrm{d}\rho\right|^2 \quad \text{(S4)}$$

where, J_0 is a zero-order Bessel function, $k=2\pi/\lambda$ is the wave number associated

with the wavelength λ, N_a is the numerical aperture, M is the magnification, ρ is the normalized pupil radius, and ϕ represents the aberration term, which is related to system parameters ψ, involving the thickness and refractive index of the immersion medium, cover glass, and observed sample. When these system parameters are known, the well-matched system PSF can be obtained through theoretical computation. On this basis, the classical Richardson–Lucy deconvolution can be employed to perform maximum likelihood estimation [2-4]:

$$f_{k+1}(x,y,z)=\left\{\left[\frac{g(x,y,z)}{h(x,y,z)\otimes f_k(x,y,z)}\right]\otimes h(-x,-y,-z)\right\} f_k(x,y,z) \quad \text{(S5)}$$

where $f_k(x,y,z)$ denotes the estimated object information at the k-th iteration.

S2 Workflow of PSF Decoupling and Sample Deconvolution

Figure S1 illustrates the overall workflow of the proposed PSF decoupling and deconvolution, comprising two stages: data acquisition and data processing. In the acquisition stage, a modulated sample is imaged in wide-field microscopy and CFM to obtain wide-field and modulated *z*-stacks for PSF decoupling, respectively. Besides, any observed sample of interest can be imaged in CFM to obtain the target *z*-stack for deconvolution.

The processing stage consists of three sequential steps.

In Step 1, the wide-field *z*-stack is deconvolved by using any applicable software and algorithm. Since an accessible tPSF can reasonably approximate the wide-field PSF, wide-field deconvolution can be flexibly implemented to obtain a deblurred z-stack, which serves as a sample prior. This computational prior will provide a reliable reference for the subsequent decoupling process.

In Step 2, the sample prior is used to computationally decouple the system PSF from the modulated response signal, i.e., the modulated *z*-stack. Here, the convergence property of PSF decoupling may differ from that of conventional deconvolution for image deblurring. A modified Richardson–Lucy algorithm was used to guarantee the convergence of PSF decoupling, which may directly impacts the accuracy and stability of the resulting cPSF (Fig. S2d).

In Step 3, cPSF is applied as a PSF prior to deconvolve the target *z*-stack to obtain the desired observation information. Similar to Step 1, any applicable software and algorithm is permissive. The difference is that Step 3 needs to be repeated for diverse observed samples, while the decoupling process (Steps 1 and 2) is performed one time to determine the final cPSF used for CFM.

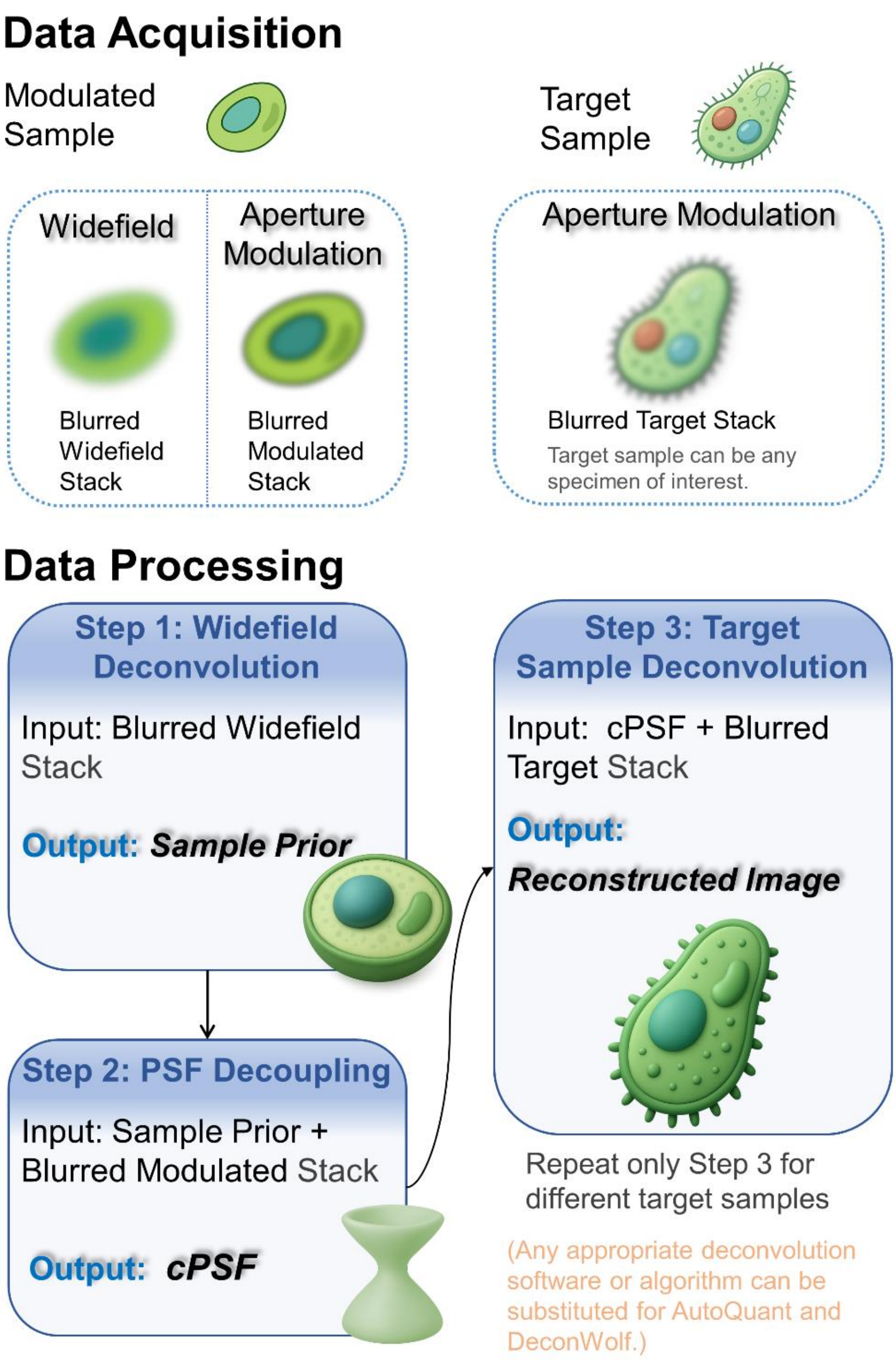

Fig. S1. Workflow of PSF decoupling and sample deconvolution. Illustration of the data acquisition and processing pipeline, including widefield deconvolution, cPSF decoupling, and target sample reconstruction using the decoupled cPSF.

S3 Convergence characteristics of PSF decoupling under iterative optimization

In the proposed PSF decoupling method, the system PSF is iteratively computed from the modulated response signal. Convergence during PSF decoupling is monitored using the relative L2 norm between adjacent iterations:

$$\frac{\|h_k - h_{k-1}\|_2}{\|h_{k-1}\|_2} < \varepsilon \quad \text{(S6)}$$

where h_k denotes the cPSF at k-th iteration, and ε is a small threshold, empirically set to 10^{-5}.

Let us take a non-modulated system as an example. A rough PSF morphology emerges after 200 iterations. However, a refined ring-like structure, which is critical for the accurate system response, stabilizes only after several thousand iterations, as shown by the evolution of cPSF morphology and the normalized cross-section intensity profiles in Figs. S2a and S2d, respectively. Figures S2b and S2c plot the SSIM and mean square error (MSE) distribution curves varying with iteration number, respectively. It can be seen that PSF decoupling tends to converge after 4000 iterations. Using a reasonable initial estimate, such as a theoretically predicted or empirically guided PSF rather than a uniform initialization, can moderately accelerate convergence. When considering the practical trade-off between accuracy and efficiency, ~4000 iterations for moderate-scale data and ~1000 iterations for large-scale data are recommended, as the cPSF beyond 1000 iterations may reach an accuracy level sufficient for practical tasks.

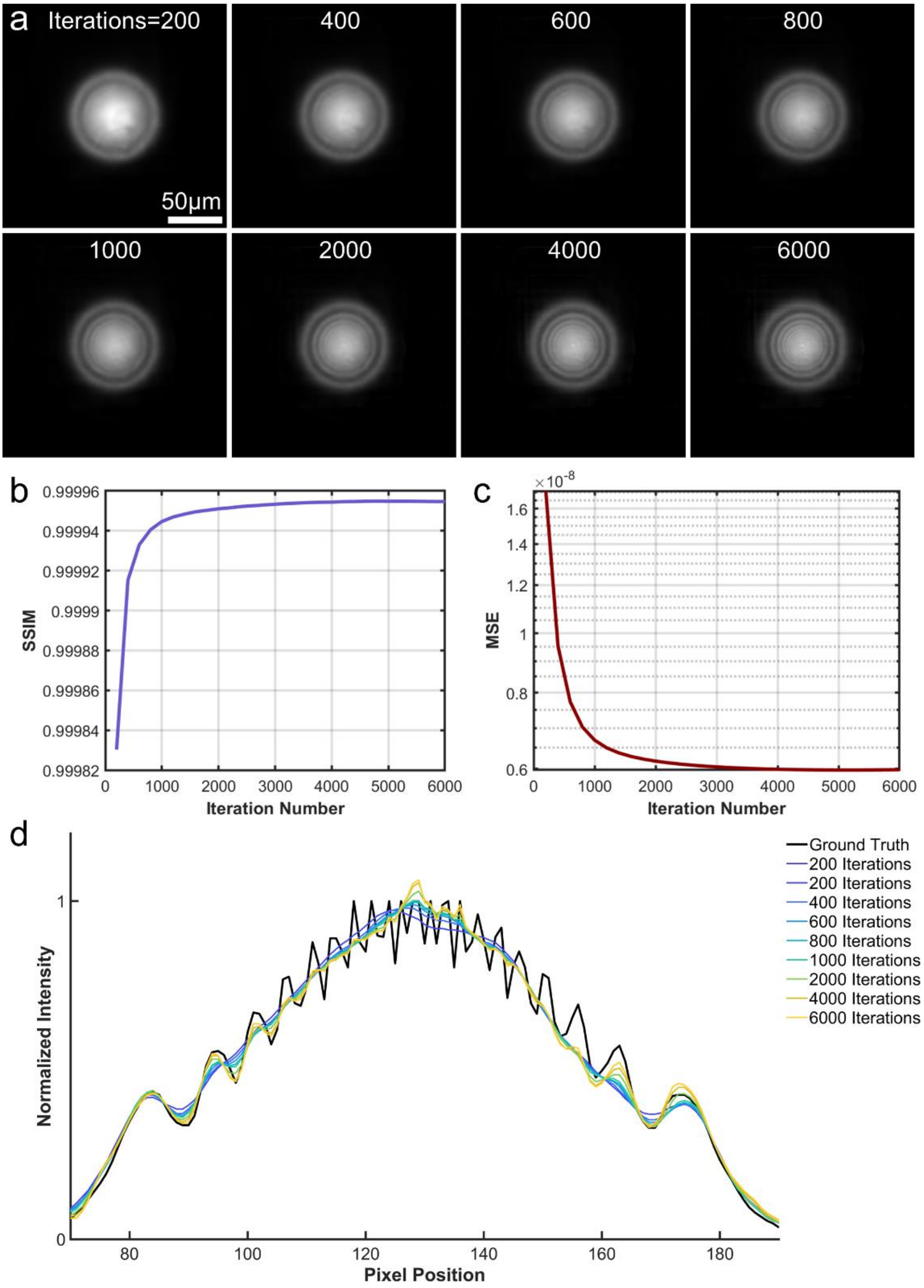

Fig. S2. Convergence behavior of the PSF decoupling algorithm under different iteration numbers. **a** evolution of the decoupled PSF (cPSF) morphology computed at iteration counts ranging from 200 to 6,000; **b** structural similarity index (SSIM) versus iteration number; **c** mean squared error (MSE, log scale) versus iteration number; **d** normalized cross-sectional intensity profiles of the cPSFs compared with the ground truth.

S4 PSF measurement for CFM

500nm-size fluorescent microspheres were used to measure the system PSF of CFM (20×/0.75) under annular aperture modulation. Figures S3a and S3b show a 16-bit single-layer image acquired with a long exposure time of 4000 ms and the local enlarged view of an individual microsphere at the region of interest, respectively. Despite employing a high-sensitivity sCMOS camera with a long exposure time to enhance the SNR of images, the grayscale distribution range was only in (5700, 18300), and the detection signal encountered substantial noise contamination, making it difficult to extract reliable PSF information. In cases where the fluorescent microspheres are unevenly distributed or densely packed, their signals will interfere with each other, resulting in inaccurate PSF estimation. Figure S3c shows the *x-y* slices corresponding to the region in Fig. S3b at various axial depths. It can be seen that the fluorescence signal decays rapidly with the increasing depth. The effective imaging range is approximately 10μm, which significantly limits the volumetric imaging performance of the fluorescence microscope.

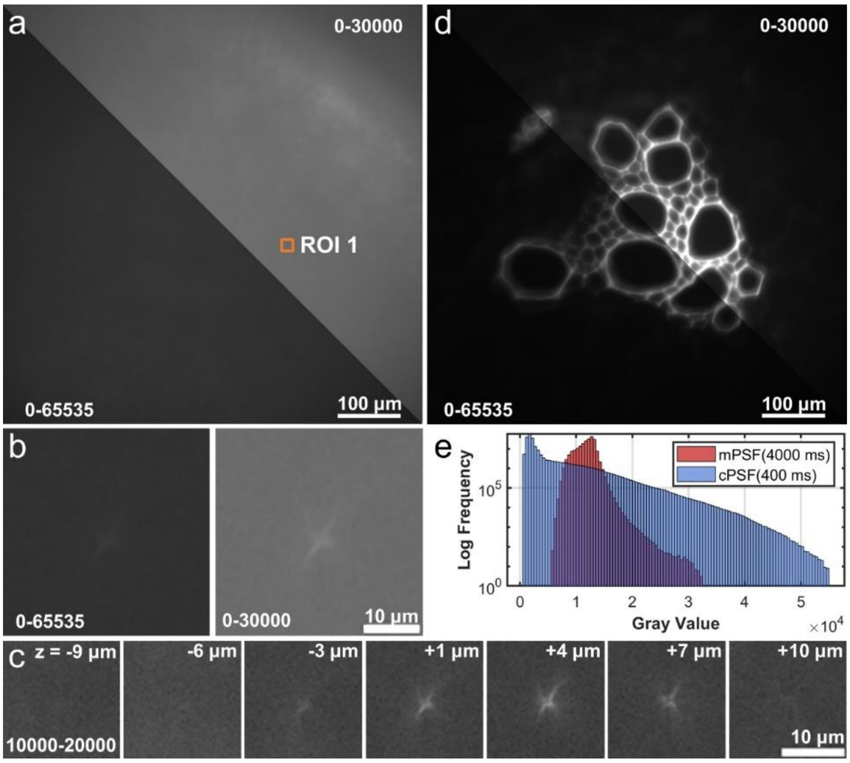

Fig. S3. PSF measurement result. **a** Single-layer image of fluorescent microspheres acquired with a long exposure time of 4000 ms, with grayscale display ranges of 0 – 30,000 (top right) and 0 – 65,535 (bottom left); **b** local enlarged views of an individual microsphere at ROI in **a**; **c** *x-y* slices corresponding to the region in **b** at various axial depths; **d** single-layer image captured with an exposure time of 400 ms, using a sunflower

stem sample as a modulated sample; **e** grayscale histogram comparison between images in **a** and **d**.

In contrast, using a sample modulator for PSF modulation and decoupling in CFM can effectively overcome limitations of the imaging depth and extraction accuracy in PSF measurement. Conventional samples with continuous structures and high-contrast stimulated emission properties, such as the sunflower stem sample shown in Fig. S3d, can be employed to achieve this purpose. Figure S3e compares the grayscale histogram distributions of sub-diffraction limit particles and the modulated sample. The modulated sample images with a high SNR could be obtained with an exposure time of only 400 ms, and the grayscale distribution range spanned (600, 44800).

S5 Cross-section distributions of PSF

Figures S4a and S4b show axial *x*-*y* slices of mPSF and cPSF across a depth range of 48 μm, corresponding to Figs. 1g and 1h, respectively. As depth increases, mPSF was progressively blurred and accompanied by irregular scattered patterns. In comparison, cPSF maintained more significant system-specific features and consistently higher SNR.

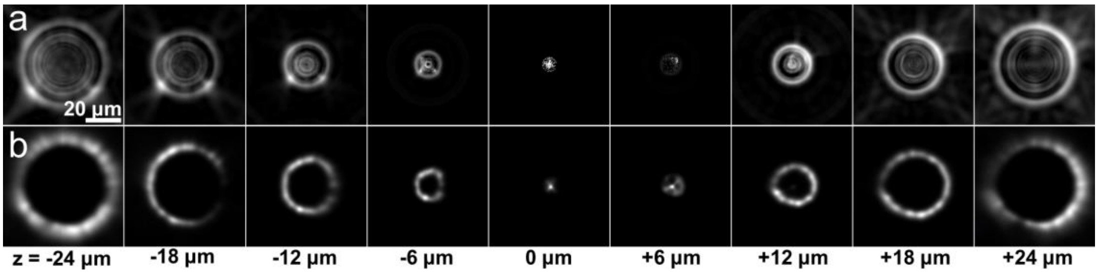

Fig. S4. PSF of CFM. Axial *x*-*y* slices of **a** mPSF and **b** cPSF across a depth range of 48 μm.

S6 PSF decoupling and deconvolution under various optical modulations

tPSF under various optical modulation modes was computed as a reference for analyzing and evaluating BD and the proposed PSF decoupling method. Under annular aperture modulation (Fig. S5a), tPSF clearly exhibits a ring-shape distribution, as shown by the axial x-y slices and 3D profile in Figs. S5b and S5c, respectively. However, bPSF deviates from such distribution, while cPSF accurately obtains the system response characteristic. The PSF accuracy will directly influence the deblurring performance, as demonstrated by the deconvolution results in Fig. S5d. The structural similarity index measure (SSIM) of the raw image and the deconvolution results using tPSF, bPSF, and cPSF with respect to the ground truth are 0.2179, 0.9399, 0.6130, and 0.9314, respectively. Furthermore, phase modulation (Fig. S5e) was introduced by applying random phase disturbance on the pupil to simulate the complex imaging aberration caused by optical path mismatch and medium scattering disturbance. tPSF under random phase modulation exhibits an asymmetric random distribution, as shown in Fig. S5f. Because BD is difficult to accurately characterize the impact of the complex aberration, the recovered object information has obvious errors, as illustrated in Fig. S5d. In contrast, through adaptive optimization in the computational imaging strategy, cPSF can effectively reflect the characteristics of random phase modulation, and the corresponding deconvolution result exhibits significantly improved accuracy. Similarly, SSIM values of the raw image and tPSF-, bPSF-, and cPSF-based deconvolution results with respect to the ground truth are 0.2232, 0.8949, 0.5154, and 0.9018, respectively. It can be seen that SSIM from cPSF is considerably high, which is close to that from tPSF. This highlights that PSF decoupling is well-suited for CFM in the presence of complex optical modulation or disturbances.

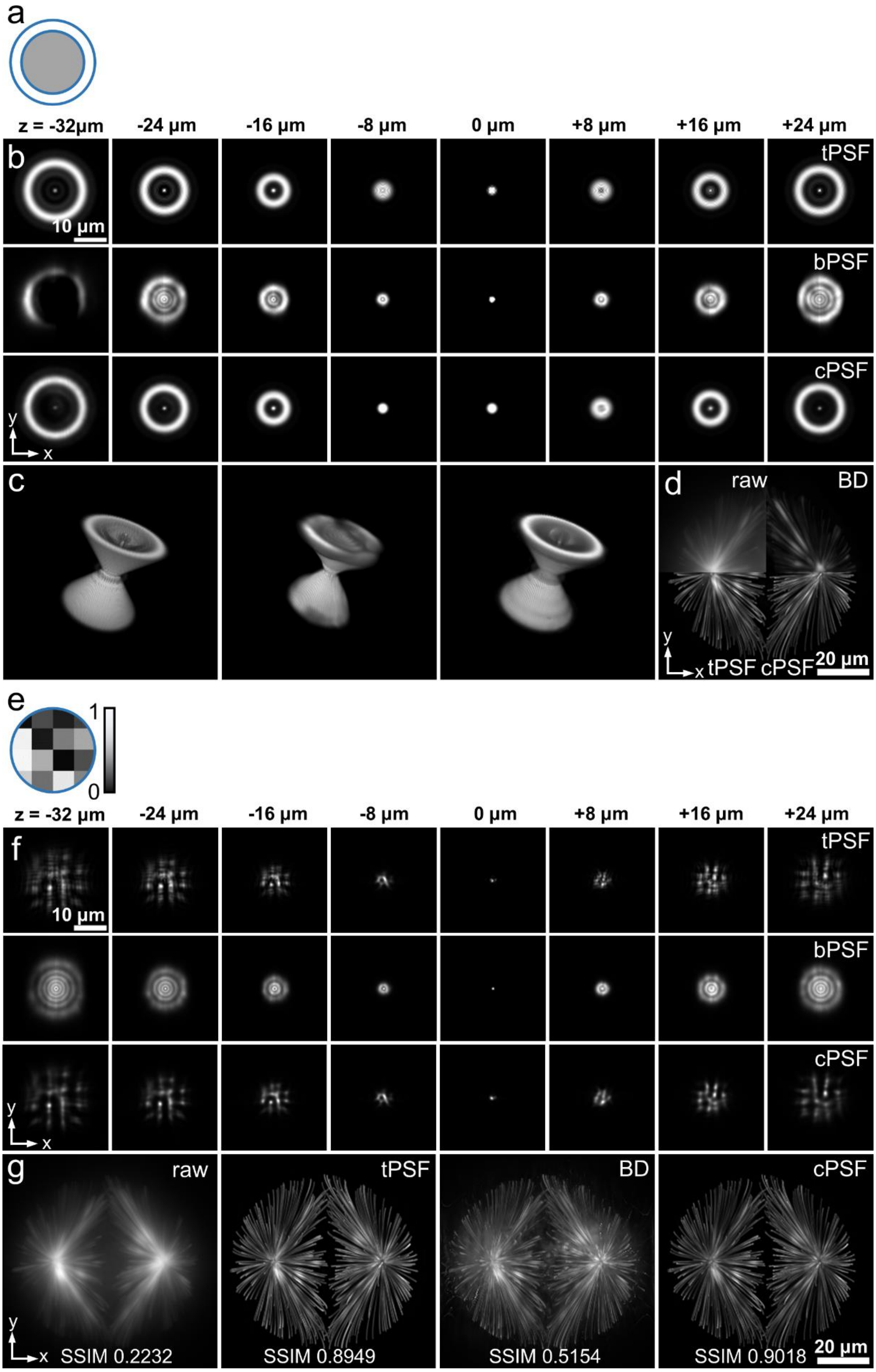

Fig. S5. PSF decoupling and deconvolution. **a** Schematic diagram of annular aperture with an obstruction ratio of 0.3; **b** axial *x*-*y* slices and **c** 3D profiles of tPSF, bPSF, and cPSF, and **d** MIP of deconvolution results under annular aperture modulation. **e** schematic diagram of random phase modulation; **f** axial *x*-*y* slices of tPSF, bPSF, and cPSF, and **g** MIPs of raw image and deconvolution results under random phase modulation.

S7 Additional performance comparison of CFM

A mouse brain section sample with a thickness of 10 μm was imaged to further demonstrate the advantages of the proposed method. Figure S6a shows the pseudo-color depth maps of deconvolution results obtained by tPSF and cPSF. The former exhibits a depth range of ~50 μm, where axial artifacts originating from system characterization mismatches severely affect object structure discrimination. In contrast, the latter demonstrates a well-contrasted depth distribution, where the volumetric reconstruction artifact is suppressed strongly. Local enlarged views of the depth map and *x*-*y* slice at different ROIs are shown in Figs. S6b and S6c, respectively. Figures S6d and S6e plot the cross-sectional intensity distribution curves related to the regions marked by the white dashed lines in Fig. S6c. From the comparison of the raw image and the deconvolution results using tPSF, mPSF, and cPSF, it can be seen that cPSF consistently achieves superior structure localization and resolution in both lateral and axial dimensions. This highlights the ability of PSF decoupling to enable accurate characterization of system response and high-fidelity volumetric imaging, particularly against scattering from biological tissue.

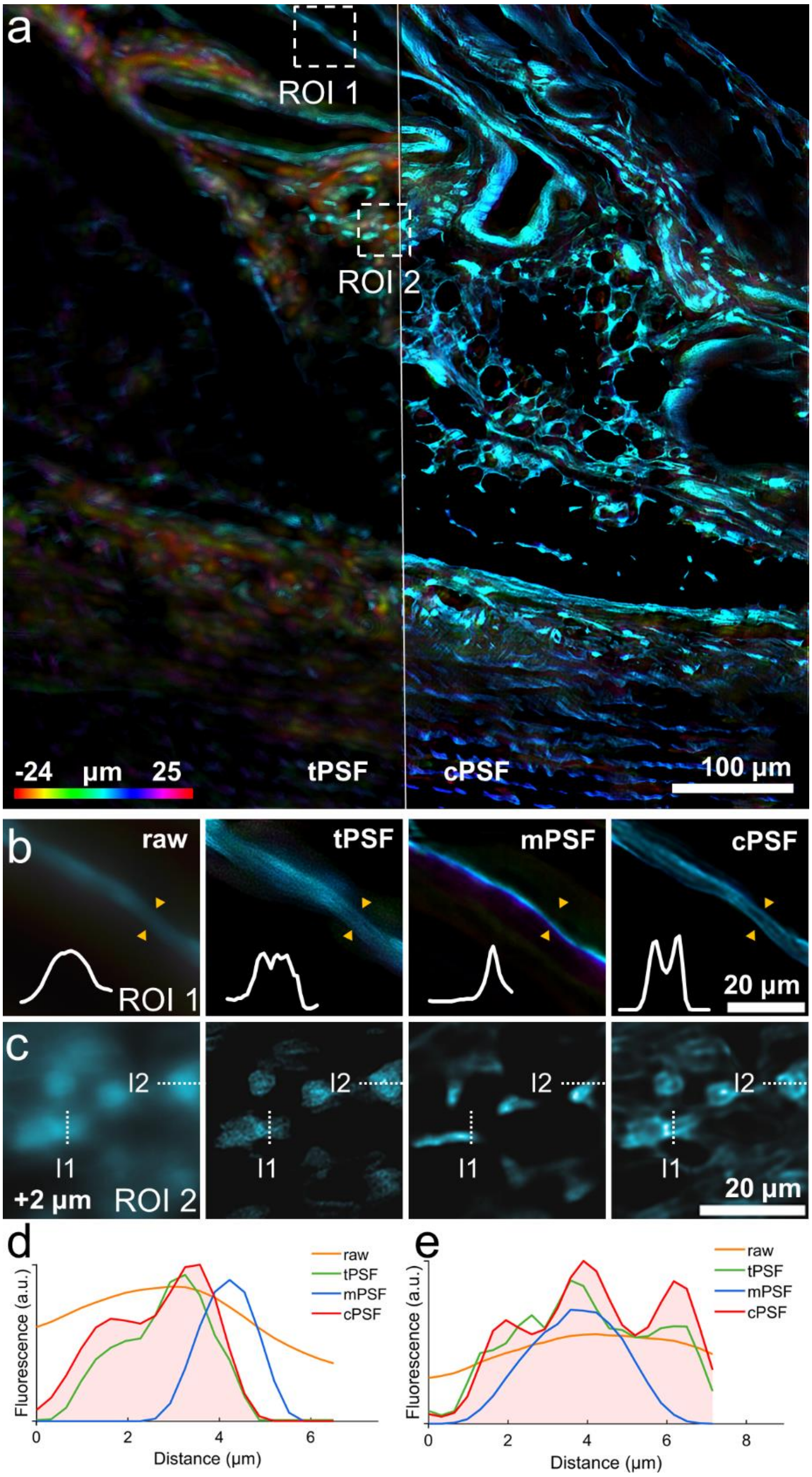

Fig. S6. Additional performance comparison in CFM. **a** Pseudo-color depth maps of the tPSF- and cPSF-based deconvolution results of a mouse brain section sample, **b**, **c** local enlarged views of the depth map and *x*-*y* slice at different ROIs, respectively, **d**, **e** cross-sectional intensity distribution curves related to regions marked by white dashed lines l1 and l2 in (**c**), comparing the raw image and deconvolution results using tPSF, mPSF, and cPSF.

S8 PSF decoupling across fluorescence channels

Using a banana stem sample with a thickness of 14 μm simultaneously as modulated and observed sample, three cPSFs were obtained from different fluorescence channels with excitation/emission wavelengths of 360/460 nm (blue), 490/530 nm (green), and 550/620 nm (red), as shown by representative *x-y* slices at different axial positions, central y-z slices, and 3D profiles in Figs. S7a – S7c, respectively. All cPSFs exhibit highly similar toroidal lateral profiles and cone-like axial structures. Table S1 lists the SSIM values among these cPSFs, all of which exceed 0.99, confirming strong agreement across channels.

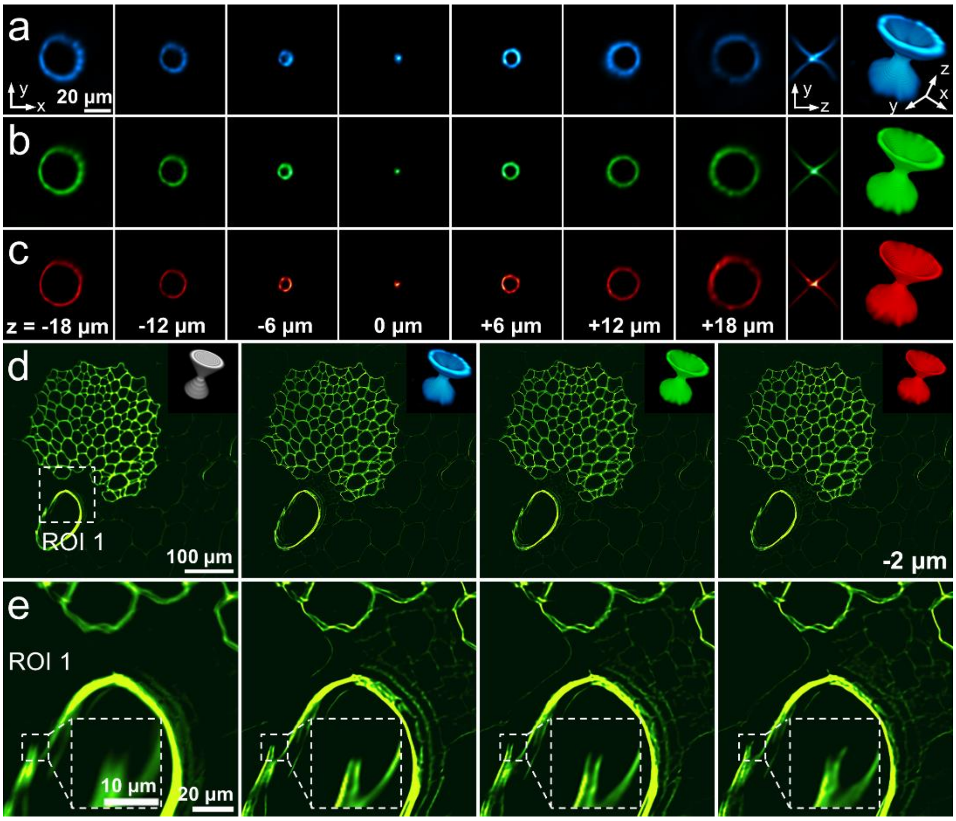

Fig. S7. PSF decoupling and deconvolution across fluorescence channels. By using a banana stem sample simultaneously as modulated and observed sample, **a**, **b**, **c** representative x-y slices at different axial positions, central y-z slices, and 3D profiles of cPSFs in three fluorescence channels (blue, green, red); **d** x-y slices of deconvolution results using tPSF and cPSFs of different channels; **e** local enlarged views of ROI in (**d**), along with secondary local enlarged views related to regions marked by white dashed wireframes.

Table S1. Structural similarity index (SSIM) between normalized cPSFs.

Channel Pair	SSIM
Blue vs. Green	0.9984
Green vs. Red	0.9990
Blue vs. Red	0.9976

The sample was observed in green channel. Figure S7d shows the *x*-*y* slices of deconvolution results using tPSF and cPSFs from different channels, and Fig. S7e shows local enlarged views of ROI in Fig. S7d. It can be seen that the tPSF-based deconvolution result fails to resolve the fine fibrous strands and vascular details. In comparison, all cPSFs yielded comparable results with high contrast and high resolution. Although the green-channel cPSF produced slightly better details because of spectral matching, the slight differences in the deconvolution results of the different channels are acceptable. The structural and functional consistency of PSF decoupling in different channels suggests that a single cPSF may be applied for CFM across broadband spectrum.

S9 Multicolor CFM

Fig. S8a shows the multicolor computational fluorescence microscopic imaging results of a wheat leaf sample under annular aperture modulation. The experiment employed excitation light sources with different wavelengths to distinguish the microfibril scaffold (green) and hemicellulose (red) of the observed sample and used cPSF-based deconvolution to restore the image quality. Due to the optical defocusing, the raw images exhibit fluorescence dispersion, leading to the blurring of the grid-like microfibril structure and the aliasing of the hemicellulose signal with the background signal, which finally affects the hierarchical resolution of the cell wall. PSF decoupling and deconvolution accurately recover the cell wall contours, significantly enhancing the contrast and making the layers of the extracellular support structure more distinct. Meanwhile, the sharpness of the microfibril edges is notably improved, and the fiber arrangement becomes clearer, as shown in Fig. S8b. Figures S8c and S8d further illustrate the local detailed structures of the microfibril and hemicellulose, which cannot be effectively resolved in cases of fluorescence dispersion and imaging blur. In comparison, cPSF-based deconvolution can restore the fine grid-like microfibril structure and the starch granules, showcasing superior resolution in multicolor CFM.

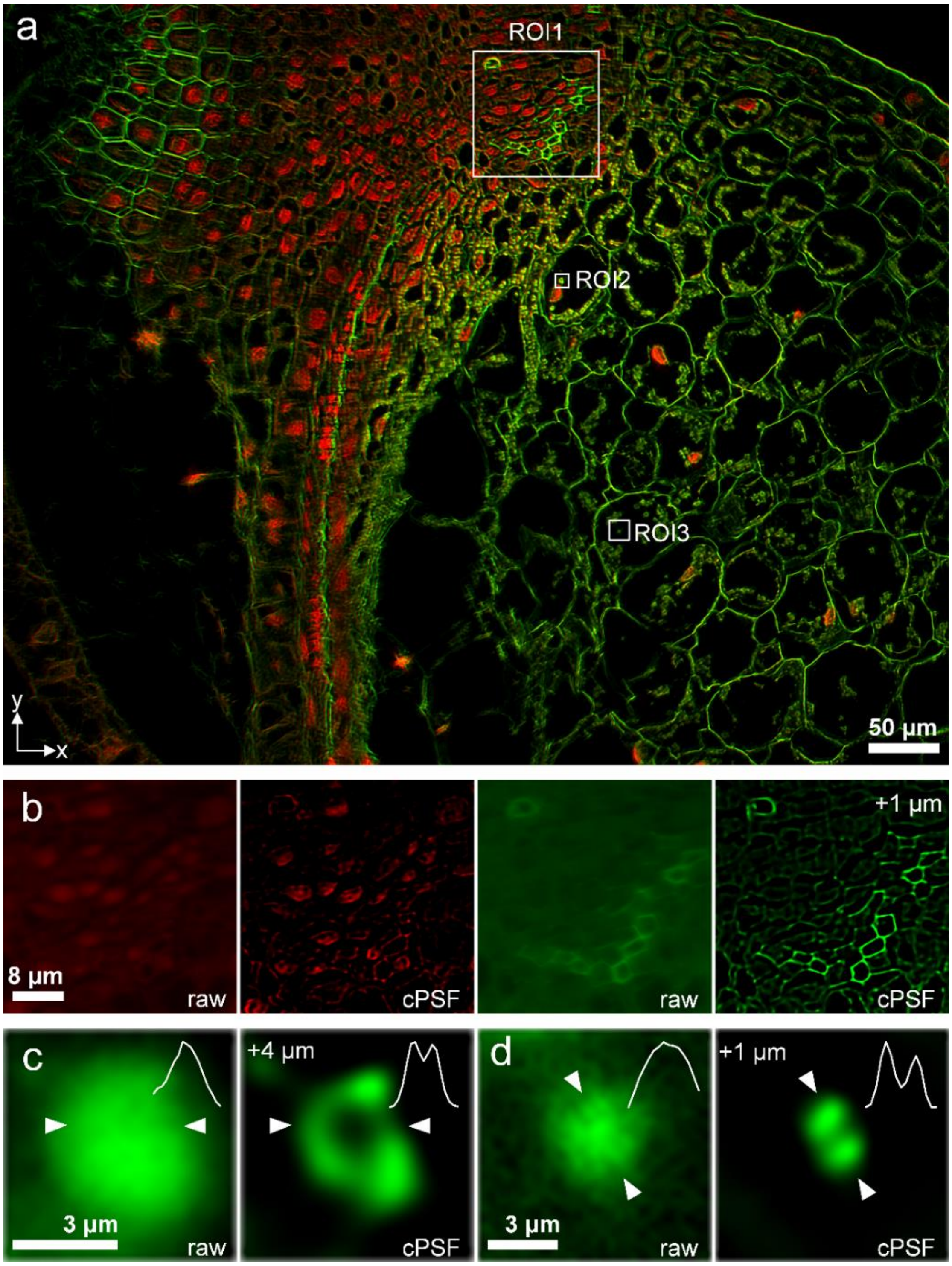

Fig. S8. Multicolor CFM result. **a** MIP of cPSF-based deconvolution result of a wheat stem sample; **b** local enlarged views of *x*-*y* slices highlighting cell wall contours (red) and grid-like microfibril structure (green); enlarged views of local detailed structures of **c** microfibril and **d** hemicellulose.

S10 Sample-specific effects in PSF decoupling

We investigated the influence of sample specificity on PSF decoupling. A wide-field microscopy with a 10×/0.4 objective was used to provide consistent imaging conditions, thus avoiding the effects introduced by system specificity. Two regular fluorescent samples with different thicknesses of 15 μm and 75 μm were used to obtain two cPSFs, labeled as cPSF1 and cPSF2, as shown by representative *x-y* slices at different axial positions and central *y-z* slices in Figs. S9a and S9b, respectively. They exhibit highly consistent overall structure, reflecting the same system response which dominates the PSF morphology. On the other hand, their subtle differences arise from the sample specificity, including light scattering, absorption, and medium heterogeneity. This observation supports the conceptual comprehensiveness of global and invariant system specificity and local and variable sample specificity in PSF decoupling.

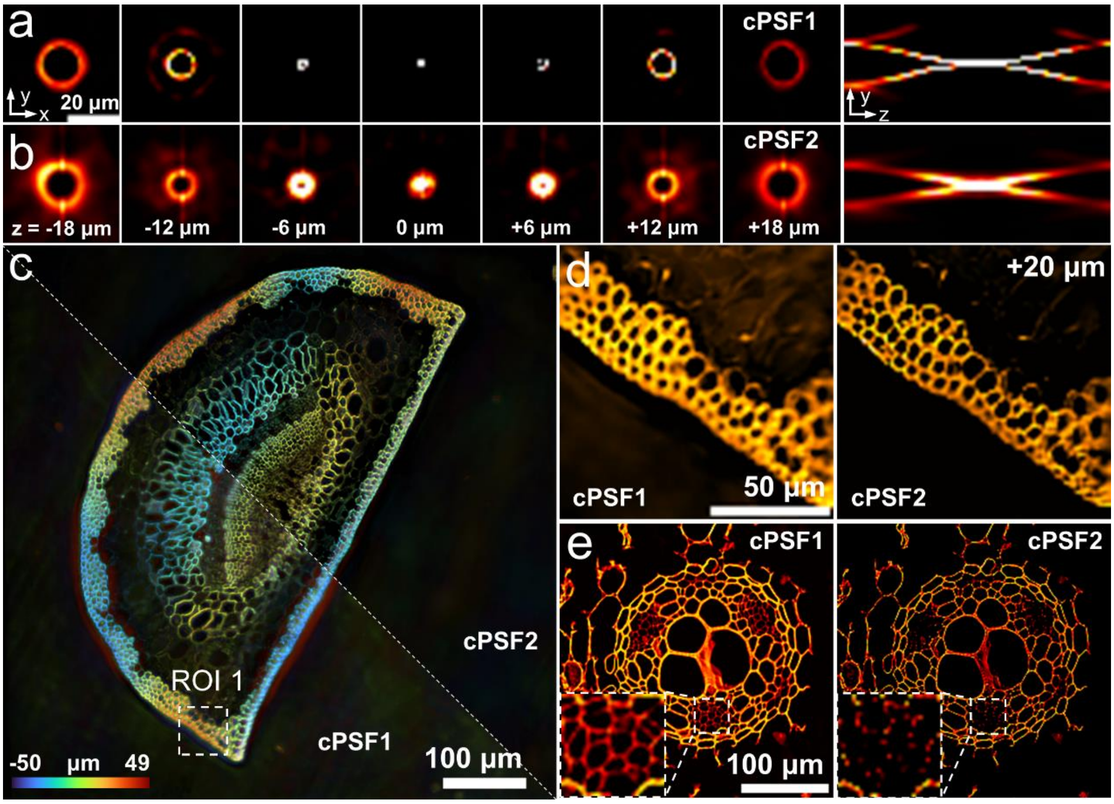

Fig. S9. Evaluation of cPSFs decoupled from modulation samples of different thicknesses and their application to deconvolution of thick and thin biological samples. **a–b,** cPSFs decoupled from a 15 μm-thick modulation sample (**a**) and a 75 μm-thick modulation sample (**b**); each row shows axial slices spanning −18 μm to +18 μm and the corresponding central **y–z** cross-section on the right, illustrating the structural differences induced by modulation sample thickness; **c–d,** deconvolution of an 80 μm-thick pine needle transverse section using cPSF1 and cPSF2, with depth encoded by color in (**c**) and enlarged views of ROI 1 at +20 μm shown in (**d**); **e,** deconvolution results of a 10 μm-thick buttercup root cross-section using the same two cPSFs, with further magnified insets highlighting fine structural details within the dashed regions.

The two cPSFs were then applied to observe a pine needle section sample with a thickness of 80 μm to assess their cross-applicability. Figures S9c and S9d show the pseudo-color depth maps and local enlarged views of *x*-*y* slices of the deconvolution results, respectively. It can be seen that the result from cPSF2 recovers finer layered structures with better artifact suppression and higher contrast in deeper regions, while cPSF1 produces slight depth smearing. Furthermore, a thin buttercup root section sample with a thickness of 10 μm was imaged, as shown by the central *x*-*y* slices of the deconvolution results respectively using the two cPSFs in Fig. S9e. In this case, cPSF1 offers slightly better performance in resolving fine features and internal fibrous details. In contrast, the result from cPSF2 tends to be more sharpened but slightly less sensitive to ultrafine structures.

Consequently, cPSF obtained from a sample modulator can be transferable to various observed samples, offering consistent imaging quality. CFM performance may be improved when the modulated sample used for PSF decoupling closely matches the observed samples. It reinforces that system specificity dominates the core PSF structure, while sample specificity contributes to fine adjustment for performance optimization.

References

1. Gibson, S. F. & Lanni, F. Experimental test of an analytical model of aberration in an oil-immersion objective lens used in three-dimensional light microscopy. *J. Opt. Soc. Am. A* **8**, 1601-1613 (1991).

2. Richardson, W. H. Bayesian-based iterative method of image restoration. *J. Opt. Soc. Am.* **62**, 55-59 (1972).

3. Lucy, L. B. An iterative technique for the rectification of observed distributions. *Astron. J.* **79**, 745 (1974).

4. Sarder, P. & Nehorai, A. Deconvolution methods for 3-D fluorescence microscopy images. *IEEE Signal Process. Mag.* **23**, 32-45 (2006).
